# Changes in health related quality of life 3 months after an acute coronary syndrome

**DOI:** 10.1186/1471-2458-6-18

**Published:** 2006-01-27

**Authors:** Inmaculada I Failde, Maria M Soto

**Affiliations:** 1 Area de Medicina Preventiva y Salud Pública. Universidad de Cádiz. Spain; 2 Hospital Universitario "Puerta del Mar". Cádiz. Spain

## Abstract

**Background:**

The aim of the study was to identify the changes in Health Related Quality of Life (HRQL) 3 months after discharge from hospital, in patients who have had an acute coronary episode, and to determine the clinical and sociodemographic variables that explain those changes.

**Methods:**

HRQL was assessed in 132 patients while they were admitted to the hospital and at 3 months after discharge, using the SF-36 health questionnaire. To identify the variables associated with the change, multiple linear regression models were constructed for two summary dimensions of the SF-36 (PCS and MCS) taking the change in the score of the dimension as dependent variable.

**Results:**

There were no significant differences between the patients who completed the monitoring (n = 76) and those who were dropped out. After three months, a significant decrease was observed in the dimensions of physical functioning, general health, vitality, and Physical Summary Component (PCS). The variables revascularisation, age, and the interaction between previous history of coronary heart disease (CHD) and the presence of one or more risk factors explained 16.6% of the decrease in the PCS. The decrease in the PCS was 6.4 points less in the patients who had undergone revascularisation, 0.2 points less for each year of age, and 4.7 points less in the patients who had antecedents of the illness as well as one or more risk factors.

**Conclusion:**

The dimensions most affected at three months after an acute coronary episode were those related to the physical component. Undergoing revascularisation improved the PCS in patients, but in the younger patients and those without personal antecedents or risk factors, the PCS was affected more, perhaps due to greater expectations for recovery in these patients.

## Background

Although the traditional measures of mortality and morbidity are frequently used as primary end points in coronary heart disease (CHD), they have limitations because they require prolonged periods of observations and are expensive to measure.

Despite the obvious value of these measures, others like health related quality life (HRQL) are considered of great importance. [1]. HRQL seems to be useful for defining health both in terms of how individuals feel (distress and well-being) and in terms of how they evaluate their health and prospects for the future [2]. Several general and disease-specific instruments have been developed to assess different dimensions of HRQL in coronary patients [3,4], with physical and mental health as their principal component [5].

Measuring changes in population HRQL is important for assessing interventions and predicting needs for social care. The focus of attention in the immediate period following a cardiac attack is generally the physical functioning, but following discharge from hospital and in the longer term, general health, vitality, social and emotional functions could be at least as important. [6].

Where studies are available, the impact on HRQL in the short and medium term after the cardiac attack appears variable, and the results are limited to populations of patients receiving specific treatment interventions. [7].

Brown et al [6] found lower scores on HRQL 4 years after myocardial infarction, compared with the community normative score, and Brink et al [8] found similar results both for the physical and the mental component 5 months after an acute myocardial infarction (AMI). On the other hand, Beck et al [7] found that, although mean scores are slightly lower at 6 months than at baseline, they did not change over the course of follow-up.

The effect of the sociodemographic variables on the HRQL in coronary patients has been described by several authors who have observed a lower HRQL in women and in subjects of low social class [9,10]. Similarly differences in HRQL have been observed in function of the presence of cardiovascular risk factors or of clinical manifestations of the disease [11,12].

Furthermore, coronary heart disease has frequently been associated with moderate to severe mental disorders, and anxiety and depression has been related with worse HRQL in these patients [13].

Despite all the preceding considerations, little is known in follow-up studies about the effect that the sociodemographic, clinical and psychological variables have on the HRQL after a coronary attack. We therefore carried out this study in order to identify the changes in HRQL at 3 months after discharge from hospital, and to identify the key clinical, demographic and psychological characteristics of patients associated with these changes.

We set up the hypothesis that physical functioning is the dimension of the HRQL showing the largest decrease after 3 months of follow-up and that age, and the clinical and mental health variables are those most closely related to these changes.

## Methods

A follow-up study was carried out in the cardiology unit of a University Hospital in the south of Spain (850 beds), where 185 consecutive patients admitted for a suspected acute episode of coronary heart disease were identified. 132 of the patients were diagnosed with acute myocardial infarction (AMI) or unstable angina on the basis of clinical, biochemical and electrocardiographic criteria described elsewhere [14] and were included in the study. Patients with non-ischemic or non-cardiological precordial pain were excluded.

The number of patients studied was based on the sample size calculated to detect a minimum difference of 5 points (α = 0.05; 1-β = 0.80) in the mean HRQL scores between the two groups of patients with AMI and unstable angina [15,16].

Baseline information (obtained 3–7 days after admission, when the patient was clinically stable) and 3 months after discharge, was obtained by a single previously-trained interviewer, who was not the cardiologist who made the clinical evaluation. Before inclusion, all the patients were asked for their informed consent and all agreed to participate (n = 132).

The ethical basis of the study was approved by the Research Committee of the Hospital.

Sociodemographic and clinical information was obtained from a structured questionnaire and from the patients' clinical records, and the Spanish Society of Epidemiology (SSE) classification [17] was used to determine social class. Cardiovascular risk factors (use of tobacco, hypertension, obesity and diabetes) and clinical information of the patients (previous history of CHD, revascularisation, ejection fraction, number of vessels obstructed, re-hospitalisation during follow-up, co-morbidity, and diagnostic group) were considered to be present when they were explicitly stated in the clinical records or in the patient's discharge report; and the existence of comorbidity other than cardiovascular risk factors was considered if another chronic pathologies (of digestive, respiratory, osteomuscular, neurological or other character) also figured in the patients' clinical records.

Health-related quality of life was assessed using the eight specific and the two Physical and Mental Components Summaries (PCS and MCS) of the SF-36 health questionnaire. For each of the dimensions of the questionnaire (PF: physical functioning; RP: physical role; BP: body pain; GH: general health; VT: vitality; SF: social functioning; RE: emotional role; MH: mental health), the items were coded, aggregated and transformed into a scale from 0 (the worst state of health for that dimension) to 100 (the best state of health). The summary indices (PCS and MCS) were calculated by standardizing each of the dimensions using the means and standard deviations of the Spanish population for their subsequent aggregation and transformation [18].

Mental health was measured using the GHQ-28 (General Health Questionnaire), an instrument developed as a method of screening to detect psychiatric non-psychotic disorders. The 28-item version was translated into Spanish and validated by Lobo et al [19] and it has already been validated as a means of detecting problems in cardiology patients [20]. This questionnaire consists of 28 items grouped into four sub-scales of 7 items each, providing additional information on: somatic symptoms of psychological origin (scale A); Anxiety or distress (scale B); Social dysfunction in every-day activities (scale C); Depression (scale D).

The score in the scale runs from 0 to 28 points, in which a higher score indicates a higher probability of mental disorders. The cut-off point recommended for the questionnaire is ≥6 points, thus providing a sensitivity of 76.9% and a specificity of 90.2% [19].

### Statistical analysis

To compare the sociodemographic and clinical characteristic of the patients at onset and 3 months alter discharge, the chi-squared and Pearson tests were done.

An analysis of the raw scores using the t-test for repeated measurements and a stratification analysis by the clinical and sociodemographic variables studied were performed for this study of the changes observed in the different dimensions of the SF-36 during the follow-up. A result was considered statistically significant if the p value was < 0.05

Multiple linear regression models were used to identify factors influencing the variance of the HRQL score 3 months after discharge, for the PCS and MCS of the SF-36. The change in HRQL scores was used as dependent variable (baseline score – 3 months score) and the independent variables to be included in the two models (age, sex, social class, GHQ-28 score, previous history of CHD, comorbidity, risk factors, revascularisation, re-hospitalisation and number of vessels obstructed) were selected following a stepwise method.

A negative result in the dependent variable reflects an improvement in HRQL during follow-up, and a negative β coefficient in the model indicates a decrease in the difference of the scores obtained after 3 months of follow-up, with respect to the initial scores.

The analysis was performed using the SPSS.v10 program.

## Results

The results presented in this paper correspond to those obtained at the start and after 3 months of follow-up, of a group of patients who have suffered an acute coronary episode, and forms part of a wider study with a follow-up period of 6 months.

Of the 132 patients who were initially included in the study, 76 (57.6%) remained after three months of follow-up. Of these 76 patients, the majority were males (81.6%), belonging to classes IV and V of the SSE classification (56.6%), and the most frequent vascular risk factors were the use of tobacco and the presence of high blood pressure. The presence of co morbidity was detected in 44.8% of the patients, and less than half of them presented a previous history of coronary heart disease. Also significant was the high percentage of patients with GHQ-28 scores of ≥6 (48.7%).

Table 1 show that there were no significant differences in socio-demographic and clinical characteristics of the responding and non-responding patients during the follow-up.

The analysis of the differences between each of the eight specific and the two summary dimensions of the SF-36 at baseline and 3 months after discharge shows a significant decrease in the scores obtained in the dimensions physical functioning, general health, vitality and in the summary physical component (Table 2). No differences were observed in the rest of the dimensions of the SF-36 (Table 2).

**Figure 1:**
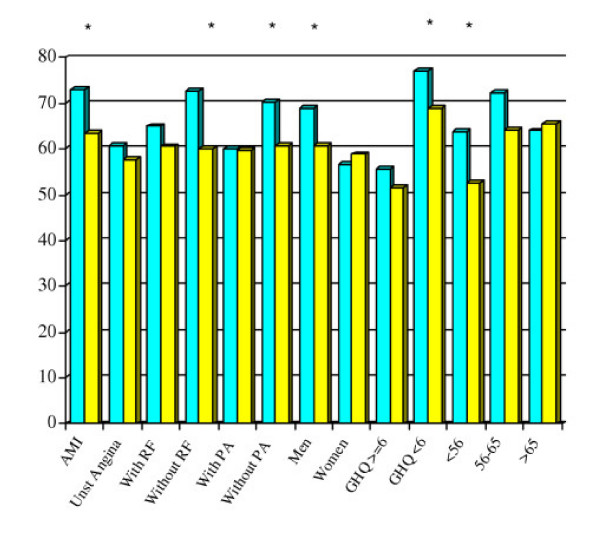
Clinical and demographic variables and vitality dimension score of the SF-36 health questionnaire at baseline and 3 months after discharge from hospital.  (Cont'd)  Baseline.  3 Months.

**Figure 2:**
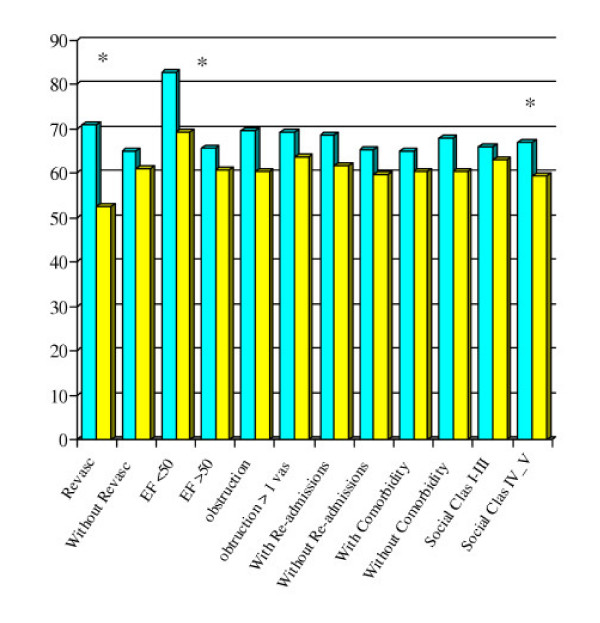
Clinical and demographic variables and vitality dimension score of the SF-36 health questionnaire at baseline and 3 months after discharge from hospital. (Cont'd)  Baseline.  3 Months.

In the stratification analysis, a significant decrease was observed after 3 months of follow-up in the dimensions of physical functioning, general health and PCS in most of the variables studied (Tables 3 and 4). No changes were observed in the MCS nor in any of the dimensions comprising it, except for vitality where a decrease was observed of the score in the patients with AMI, without antecedents of CHD, without cardiovascular risk factors, males, those with lower scores in the GHQ-28, those belonging to the lowest social classes, of younger ages, those with revascularisation and with ejection fraction lower than 50 (Fig 1 and Fig 2).

In the dimension bodily pain it is notable that the scores at 3 months of follow-up increased in the patients with revascularisation, and the scores decreased in the women. (Table 3 and 4)

Only the multiple regression model constructed with the PCS as the dependent variable is considered relevant for this analysis of the variables associated with the changes occurring during the follow-up. This model shows revascularisation performed during the follow-up, age and the interaction between personal history of CHD and the presence of one or more risk factors to be explanatory variables of the change in the PCS. Thus, the decrease in the PCS at 3 months was 6.4 points less in those patients submitted to a revascularisation, and 0.2 points less for each year of age. Further, although the presence of one or more risk factors or of antecedents of CHD does not explain the changes in the PCS, a reduced decrease (4.7 points) at 3 months is found in those patients who had personal history of the disease if they also had one or more cardiovascular risk factor (Table 5).

## Discussion

This study reveals a decrease in the summary physical component of the HRQL in coronary patients 3 months after having an acute episode of the disease, and identifies the patient characteristics associated with this change. In the study it is also demonstrated that the scores in the dimensions of the HRQL related to the mental component are maintained during the follow-up. However, these scores were low at baseline and lower than those observed in the Spanish general population [21].

These results are in agreement with those obtained by other authors such as Hemingway et al [17] and Brown et al [6], who find that the physical functioning is affected more than mental functioning. However, in patients with AMI, Beck and cols [7] find only small differences in the PCS of the SF-36 after 6 months of follow-up, and find no differences at one year; Bengtson et al [22] find significant improvement in physical health over time only in patients older than 59 years of age.

Although in our study vitality decreased after 3 months of follow-up, its effect remained hidden by the MCS of the HRQL, which showed no difference from the base score. These results are in accordance with those found by Veenstra et al. [23] and could be explained by the inclusion in the MCS of other dimensions such as social functioning, mental health and emotional role that showed no changes during the follow-up. Heller et al [24] find poor emotional health in patients with AMI or angina 6 weeks after discharge, but this dimension returns to baseline levels at 6 months. However Rubenach et al.[25] show that mental health remained poorer compared to baseline at 6 months after discharge, and attributed these differences to the effect on the summary score of the dimensions vitality and social functioning, which were low at 6 months compared to baseline values.

Among the factors associated with the change in the PCS, age, revascularisation and the interaction between antecedents of coronary heart disease and the existence of coronary risk factors were the most important. In an analysis of the factors associated with the HRQL at 6 and 12 months after discharge, Beck et al. [7] observed that age was a factor that predicted an impaired PCS in both cases, with the older patients obtaining lower scores. These results are not in accordance with those found in our study where for each year that the age increases, the PCS score improves by 0.2 points. One explanation of these results, which has been shown in other disease states, could be that the experience of the elderly and the degree of dissatisfaction felt at a given level of disability is less than in younger individuals [6,26]. Another possible explanation could be that younger patients were treated more aggressively after initial hospitalisation [7,27], making the recovery longer and affecting the HRQL more. However, this latter explanation does not appear plausible due to the results having been adjusted for the treatment received by the patients, in that study.

Revascularisation was the only clinical variable associated with change in the PCS, and no association was observed with other variables related to the severity of the disease or the administration of treatments. These results are in accordance with those observed by Veenstra at al [23]. However Beck et al [7] find a decrease in the scores of the PCS in the patients who have had revascularisation. These differences may perhaps be due to Beck and cols considering the previous revascularisation as a characteristic relating to the clinical history of the patients, as against ourselves considering revascularisation as a therapeutic measure undergone during the follow-up.

The antecedents of CHD and the presence of cardiovascular risk factors considered individually were not variables associated with the change in the PCS scores. However, those patients who met the two criteria present a PCS score at 3 months that is more favourable than their initial score. According to Steward et al [28], first-time admission patients with ischemic heart disease assessed their illness as less threatening and used different strategies for coping compared with those having multiple admissions for cardiac disease. It is also been suggested that individuals with a strong sense of coherence – an internal resource for coping that helps the person confront stressful situations – may be better prepared to cope with acute myocardial infarction [29].

Patient's sex and the existence of "probable mental disease" were not variables associated with the change in the PCS score during the follow-up, unlike the findings of other studies [10] although they were variables that predicted the PCS, and especially the MCS, at baseline [30]. In the present study, the GHQ-28 questionnaire was completed before discharge and probably this assessment did not reflect the mental health of the patients 3 months later.

One of the limitations of the study could be the use of a generic instrument for the assessment of HRQL that may be less sensitive to change than a specific instrument. However, this instrument was used because we were interested in measuring subjects' perceptions of their "overall HRQL" after a coronary attack. Furthermore, the small sample in the study could diminish the statistical power and underestimate the differences in the follow-up. [31].

Another of the limitations is that it has not been possible to calculate the minimum clinically important difference, because no subjective evaluation was made of the change in the state of health of the patient different from that of the SF-36. Nevertheless, changes of more than 12% over the initial score have been considered acceptable for the SF-36 by other authors [32], and in our results differences in the PCS of more than this magnitude are observed.

## Conclusion

Our findings suggest that in a non-selected cohort of short term survivors of a coronary attack, there is a notable effect on HRQL, especially in physical functioning, in younger patients without personal antecedents or coronary risk factors, that could go unrecognised. In addition, it demonstrates the need for complementary treatment to improve the mental component of HRQL in the patients of the study.

The identification of predictors of HRQL at the time of admission would allow physicians to identify those patients with a worse HRQL and to compare the effects of different treatments during follow-up.

Also, in view of the results obtained, the SF-36 seems to be a sensitive tool for HRQL assessment in coronary patients, and provides valuable information not identified in routine clinical evaluation.

## Competing interests

The author(s) declare that they have no competing interests.

## Authors' contributions

I.F.:

1) She has made substantial contributions to conception and design of the paper,

2) She has been involved in drafting the manuscript or revising it critically for important intellectual content;

3) She has given final approval of the version to be published.

M.S.:

1) She has made substantial contributions to the collection, analysis and interpretation of data, and she has helped to draft the manuscript.

All authors read and approved the final manuscript.

## Pre-publication history

The pre-publication history for this paper can be accessed here:



## Figures and Tables

**Table 1:** Comparison of the characteristics of the patients at the start and after 3 months of follow-up.

*Sociodemographic variables*			
	ONSET n(%)	3 MONTHS n (%)	p
*Age*			0.26
<46	3 (5.4)	9(11.8)	
46–55	9(16.1)	18(23.7)	
56–65	21(37.5)	23(30.3)	
>65	23(41.0)	26(34.2)	
*Sex*			0.34
Men	41(73.2)	62(81.6)	
Women	15(26.8)	14(18.4)	
*Social class*			0.67
Housewives	12(22.2)	12(15.8)	
I and II	5(9.3)	5(6.6)	
III	8(14.8)	16(21.0)	
IV and V	29(53.7)	43(56.6)	

*Clinical variables*			
	ONSET n (%)	3 MONTHS n (%)	P

*Diagnostic group*			0.91
Acute myocardial infarction	26(46.4)	37(48.7)	
Unstable angina	30(53.6)	39(51.3)	
*GHQ-28 (initial)*			0.84
< 6	29(51.8)	39(51.3)	
≥ 6	27(48.2)	37(48.7)	
*Tobacco*			0.82
Smoker	25(45.5)	33(43.4)	
Non smoker	30(54.5)	43(56.6)	
*Diabetes*			0.53
Yes	34(61.8)	51(68.0)	
No	21(38.2)	24(31.6)	
*Hypercholesterolemia*			0.46
Yes	36(65.5)	45(59.2)	
No	19(34.5)	31(40.8)	
*High blood pressure*			0.22
Yes	23(41.8)	40(52.6)	
No	32(58.2)	36(47.4)	
*Obesity*			0.34
Yes	19(35.8)	19(25.7)	
No	34(64.2)	55(72.3)	
*Previous history of CHD.*			0.20
Yes	26(46.4)	27(35.5)	
No	30(53.6)	49(64.5)	
*Comorbidity*			0.63
Yes	22(39.3)	34(44.8)	
No	34(60.7)	42(55.2)	

**Table 2:** Changes in HRQL 3 months after discharge from the hospital

	ONSET		3 MONTHS		p
		
	Mean	SD	Mean	SD	
PF	77.3	25.77	61.4	26.69	0.000
RP	67.4	42.23	57.9	47.99	0.18
BP	65.9	30.91	69.3	30.00	0.39
GH	55.1	19.69	50.5	17.97	0.03
VT	66.7	23.58	60.5	22.36	0.04
SF	80.6	25.69	80.1	25.09	0.86
RE	62.7	46.18	60.1	48.69	0.67
MH	66.6	24.58	64.2	24.31	0.33
PCS	47.1	9.97	42.8	9.95	0.006
MCS	44.1	15.16	45.2	13.95	0.78

PF: physical functioning; RP: physical role; BP: body pain; GH: general health.

PCS: physical component summary; VT: vitality; SF: social functioning; RE: emotional role; MH: mental health; MCS: mental component summary; SD: standard deviation.

**Table 3:** PCS and physical dimensions scores of SF-36 health questionnaire at baseline and 3 months after discharge from the hospital (demographic and clinical variables)

	PF			RP			BP			GH			PCS		
	ONSET	3 MONTHS	P	ONSET	3 MONTHS	P	ONSET	3 MONTHS	P	ONSET	3 MONTHS	P	ONSET	3 MONTHS	P

*Diagnostic group*															
AMI	85.2(22.8)	66.6(26.6)	0.000	71.6(40.9)	61.5(48.1)	0.333	69.6(31.7)	77.6(26.6)	0.184	63.6(17.4)	56.3(18.6)	0.024	50.2(9.3)	45.2(10.4)	0.040
Unstable angina	69.7(26.4)	56.5(26.2)	0.001	63.5(43.6)	54.5(48.3)	0.371	62.4(30.1)	61.4(31.2)	0.855	47.2(18.5)	44.9(15.7)	0.459	44.3(9.8)	40.6(9.1)	0.080
*Risk factors*															
None	91.2(9.7)	66.2(29.2)	0.001	83.8(34.2)	47.0(49.1)	0.007	70.5(26.9)	68.0(32.7)	0.765	62.3(21.9)	53.3(15.6)	0.093	51.6(11.0)	41.3(13.2)	0.011
One or more	73.3(27.5)	60.1(26.0)	0.000	62.7(43.4)	61.0(47.6)	0.838	64.6(32.1)	69.7(29.5)	0.265	52.9(18.6)	49.6(18.6)	0.169	45.7(9.3)	43.2(8.8)	0.159
*Previous history CHD*															
No	86.5(19.9)	67.9(26.4)	0.000	75.0(38.8)	59.7(48.1)	0.086	70.3(28.7)	73.1(27.5)	0.542	58.0(19.9)	51.9(17.5)	0.010	50.6(8.9)	44.4(10.1)	0.002
Yes	60.5(27.0)	49.6(23.2)	0.042	53.7(45.3)	54.6(48.5)	0.940	57.9(33.6)	62.3(33.5)	0.564	49.8(18.5)	47.9(18.9)	0.658	40.7(8.5)	39.9(9.2)	0.836
*Sex*															
Men	80.5(24.0)	64.7(25.5)	0.000	68.5(41.7)	56.4(25.5)	0.137	63.9(31.0)	71.2(29.3)	0.123	56.1(19.5)	49.8(16.9)	0.005	47.1(10.2)	42.9(9.9)	0.014
Women	62.8(29.1)	47.1(28.2)	0.032	62.5(45.7)	64.3(49.7)	0.908	74.7(29.8)	61.0(32.8)	0.007	50.6(20.4)	53.3(22.3)	0.683	46.8(9.1)	42.3(10.3)	0.263
*Age*															
<56	82.8(24.9)	68.3(23.0)	0.000	69.4(40.6)	38.9(48.2)	0.023	60.1(32.6)	66.6(30.6)	0.342	52.7(20.2)	49.5(17.3)	0.225	48.3(10.6)	42.4(9.7)	0.006
56–65	83.4(19.1)	63.7(28.4)	0.002	64.1(45.1)	65.2(48.7)	0.927	72.7(28.4)	64.7(36.8)	0.276	53.4(20.8)	50.4(21.9)	0.559	47.4(9.1)	44.2(11.3)	0.302
> 65	66.1(28.7)	52.3(27.0)	0.013	68.3(42.7)	71.1(42.2)	0.806	66.0(31.1)	76.2(21.4)	0.135	58.8(18.3)	51.7(15.5)	0.041	44.5(9.8)	42.3(8.9)	0.324
*Social class*															
I, II, III	77.8(24.1)	66.9(23.0)	0.016	61.9(43.0)	64.2(45.8)	0.871	69.2(28.3)	71.0(27.7)	0.819	56.8(21.2)	52.4(14.7)	0.208	47.2(10.2)	44.9(8.7)	0.295
IV, V, housewives	77.1(26.5)	59.3(27.8)	0.000	69.5(42.1)	55.4(48.9)	0.087	64.6(31.9)	68.6(31.0)	0.397	54.4(19.2)	49.7(19.1)	0.084	46.5(9.8)	42.1(10.2)	0.014

**Table 4:** PCS and physical dimensions scores of SF-36 health questionnaire. Baseline and 3 months after discharge from the hospital. (Clinical variables)

	PF			RP			BP			GH			PCS		
	ONSET	3 MONTHS	p	ONSET	3 MONTHS	p	ONSET	3 MONTHS	p	ONSET	3 MONTHS	p	ONSET	3 MONTHS	p

*Revascularisation*															
No	75.2(26.9)	59.7(27.6)	0.000	68.2(42.3)	60.1(47.3)	0.338	70.1(31.3)	67.7(29.4)	0.601	55.1(21.4)	49.0(17.9)	0.022	47.2(9.9)	42.7(8.6)	0.004
Yes	81.7(19.7)	69.6(17.8)	0.134	76.8(38.5)	48.2(50.4)	0.063	50.6(25.1)	75.2(30.9)	0.015	46.7(14.9)	51.2(17.8)	0.286	43.8(7.9)	44.4(10.6)	0.842
*Ejection fraction*															
< 50	77.8(28.8)	61.4(32.1)	0.099	50.0(50.0)	75.0(43.3)	0.251	73.1(35.4)	77.0(28.6)	0.714	60.7(25.0)	49.4(19.2)	0.211	44.8(15.3)	42.9(8.8)	0.639
≥ 50	78.6(26.1)	63.6(25.4)	0.000	72.2(39.4)	58.1(48.2)	0.191	66.4(28.0)	68.0(31.6)	0.787	51.4(19.6)	51.6(19.3)	0.932	46.3(8.3)	43.7(8.5)	0.162
*Number of vessels*															
One vessel	79.7(26.6)	68.4(25.5)	0.028	80.6(36.1)	46.5(48.3)	0.009	71.9(29.3)	77.3(27.4)	0.443	57.3(21.2)	50.5(17.9)	0.017	48.3(8.5)	43.6(8.2)	0.035
More than one vessel	77.8(25.6)	63.7(25.9)	0.002	55.3(45.3)	69.6(45.8)	0.232	59.7(30.8)	67.2(31.4)	0.262	51.1(16.4)	52.3(19.3)	0.730	43.6(10.3)	43.6(9.9)	0.996
*Re-admissions*															
None	77.9(25.7)	64.8(27.5)	0.000	72.6(39.6)	55.2(48.3)	0.060	65.8(30.9)	70.2(31.1)	0.392	54.8(19.6)	49.3(17.8)	0.022	48.1(9.2)	43.4(9.3)	0.003
Some	77.7(25.2)	58.7(26.2)	0.001	62.1(46.5)	62.9(47.5)	0.942	68.6(31.8)	68.9(28.4)	0.968	53.4(20.5)	51.3(18.6)	0.621	45.2(11.2)	42.4(11.0)	0.329
*Comorbidity*															
No	83.2(22.6)	66.5(24.6)	0.000	66.6(41.5)	56.5(49.1)	0.283	64.8(31.0)	68.5(30.8)	0.480	56.7(20.1)	54.3(16.3)	0.383	46.9(9.3)	44.3(9.4)	0.094
Yes	70.0(27.7)	55.1(28.1)	0.002	68.3(43.6)	59.5(47.2)	0.433	67.2(31.1)	70.2(29.3)	0.632	52.9(19.2)	45.6(18.9)	0.038	46.4(10.7)	41.0(10.2)	0.037
*GHQ-28*															
< 6	89.6(16.3)	71.8(26.5)	0.000	80.1(36.8)	70.5(45.5)	0.312	73.3(31.5)	76.7(27.1)	0.501	65.5(15.4)	57.9(15.9)	0.011	47.7(10.2)	47.1(8.5)	0.754
≥ 6	64.3(27.7)	50.5(22.4)	0.006	54.0(43.9)	44.6(47.5)	0.389	58.2(28.7)	61.5(31.2)	0.596	43.8(17.6)	42.6(16.7)	0.686	45.8(10.3)	38.4(9.1)	0.002

**Table 5:** Multiple linear regression model for the change in the physical component summary (PCS)

Coefficient of determination of the model: R^2 ^= 0.166. Significance = 0.012			
	Coefficients	Standard error	Significance
Constant	21.737	7.430	0.005
Revascularisation	-6.497	3.205	0.047
Age	-0.259	0.122	0.037
One or more risk factors* × antecedents of CHD**	-4.783	2.684	0.080

*One or more risk factors: Use of tobacco, diabetes, hypercholesterolemia, high blood pressure, obesity. **CHD: coronary heart disease.

Reference categories: No revascularisation. Age in years. No risk factor. No antecedents of CHD.
